# Professional Quackery

**Published:** 1855-03

**Authors:** 


### PROFESSIONAL QUACKERY.

Various forms of quackery are constantly obtaining among those who call themselves regular physicians. Our attention has been called to a few which demand notice. We cannot doubt that these irregularities often occur from ignorance or a misunderstanding of certain principles which are clearly laid down in the code of medical ethics. Some of these practices, however, involve the physician in a breach of the plainest laws of morality. We should suppose that the impropriety and even the immorality of encouraging directly or indirectly the use of quack medicines would be obvious to any one who pretends to practice physic upon scientific principles. Some may fall into the error insensibly. Many we know find it necessary to keep a stock of drugs, and some add to their income by keeping drug stores. This may be necessary, as we have said; but, there can be no need of keeping patent medicines. Abundance of excellent formulæ may be found in the Dispensatory and other standard medical works. We know the physician who has these medicines in his own drug store, may be tempted to use them either to save himself the trouble of compounding or studying out a remedy. But there can be no excuse for such a course. The component parts of these quack remedies are a secret, and he who prescribes them, practices dishonesty both with himself and his patient—with himself because he thus gives the lie to his own profession of ability to treat disease—with his patient since he is giving him he knows not what.

By recommending or vending these nostrums also, the physician is almost as directly encouraging one of the worst kinds of quackery, one of the greatest forms of imposition. We believe that no one who has any regard for the welfare of his profession or the advancement of true science—no one who possesses a suitable degree of self-respect—no one, finally, who places before himself a proper standard of right, will do any thing which has even the appearance of countenancing this dishonest and unrighteous traffic.

Nor can any right-minded physician keep secret those means which he may have found useful in the treatment of disease. If he lays any claim to that high name which has been accorded, in all time, to the members of his profession as "friends of humanity," he will make known such modes of treatment, as chance or skill may have placed at his command, for the relief of suffering mortality. If he is actuated by motives of justice, he will endeavor, in this manner, to repay somewhat of the debt he owes to those from whose labors his own power is derived—he will feel that he has no right to reap the fruit of another's toil, unless he is willing to add all he can to the common fund of knowledge. If he has entered the field of professional action with a proper appreciation of its duties; or, if he desires to pursue his calling in the only spirit which can lead to honorable distinction, he will not let pass any opportunity of contributing his mite to the cause of medical science.

Some of those who follow legitimate medicine, countenance quackery by meeting, in consultation, irregular practitioners. We are aware that whenever a physician refuses to meet a quack at the bed-side of a patient, he is called illiberal. But of such illiberality, we should be proud to see every member of our profession possessed. It would seem as though the common sense of the people should perceive the injustice of expecting a physician to submit to such an indignity. The physician himself, is highly culpable who will consent to consult with quacks of any name. The only headway ever made by all the allied host of empiricism, has been by their constant out-cries against the regular practice—by wholesale abuse—by incessant reviling, and by unblushing misrepresentation.

They ridicule the knowledge and wisdom of those whose hairs have grown grey in pursuit of science—they laugh to scorn the toils and labors which have occupied whole lives of men really great and truly devoted to their profession—they studiously conceal whatever of good they may see, and parade in the most odious manner whatever imperfections may appear in the practice of legitimate medicine. In spite of all these things, they are astonished and horrified because regular physicians refuse to meet them in consultation! But it is actually dishonest on another account. Whenever and wherever the physician is called to practice his art, he must have a clear field, and if he consult with any, it must be with those and those only, who, acting in good faith, will do all they can to aid his efforts.

If any person has sufficient confidence in a quack to employ him he will not take the advice of a regular physician, so that the presence of the latter is useless. If the quack himself wish the other's attendance, the only supposition can be that he wishes for assistance to correct his own blundering diagnosis. But it is not our duty to instruct those who set at naught our knowledge. Our business is to cure, not to teach ignorant pretenders. We have seen some excellent remarks in the June No. of the Nashville Journal of Medicine and Surgery. A quotation is made from an article by Dr. Reese, of New York, Editor of the American Medical Gazette, in which the subject in point is illustrated by the question, whether an Evangelical Minister would meet a Mormon Elder at the bed-side of a dying person, and co-operate with him in his endeavors to prepare a soul for eternity?

Our case is even stronger than this, since the Mormon believes in the Bible, and in Jesus Christ as the Son of God; but the regular physician, who meets the quack in consultation, acts as inconsistently as the clergyman who should fraternise with the open and avowed infidel who considers or pretends to consider the religion of the Bible false. The editorial of the Nashville Journal has the following excellent sentence:—"If we would preserve self-respect, the dignity of our calling and the good of society, the gulf between the quack and the physician *must* be so wide and deep as to be utterly impassable.

There is another practice, no less reprehensible than those we have mentioned above, which we most earnestly hope to see done away with. We refer to the custom of attending on families for a fixed amount *per annum*, or as it is called, "doing *doctoring* by the year." This is well worthy of the ignorant quacks who are everywhere imposing themselves upon the public. We have heard such a practice most strongly condemned by non-medical men as dishonest, and in a high degree dishonorable. It is manifestly dishonest, since no physician can foresee how his time may be occupied. If a family depends on him for medical aid, he may be absent at that critical moment when his services are most needed. Besides, all such things injure the standing of the profession, and degrade it from the rank it should maintain. We trust our brethren will open their eyes to this evil, and not strive to compete with ignorant and unscrupulous quackery in any such matter.

If we justly appreciate the true glory of our calling, we shall always be alive to its highest and best interests, and nothing will tempt us to turn aside from that path to which a sense of propriety and honor ever point.

